# Selection and validation of reference genes for RT-qPCR analysis in potato under abiotic stress

**DOI:** 10.1186/s13007-017-0238-7

**Published:** 2017-10-16

**Authors:** Xun Tang, Ning Zhang, Huaijun Si, Alejandro Calderón-Urrea

**Affiliations:** 1 Gansu Key Laboratory of Crop Genetic and Germplasm Enhancement, Gansu Provincial Key Laboratory of Aridland Crop Science, Gansu Agricultural University, Lanzhou, 730070 People’s Republic of China; 2 College of Agronomy, Gansu Agricultural University, Lanzhou, 730070 People’s Republic of China; 3 College of Life Science and Technology, Gansu Agricultural University, Lanzhou, 730070 People’s Republic of China; 4 Department of Biology, California State University, 2555 East San Ramon Avenue, Fresno, CA 93740 USA

**Keywords:** Reference gene, Drought stress, Osmotic stress, Potato

## Abstract

**Background:**

Real-time quantitative PCR (RT-qPCR) is the most commonly used method for accurately detecting gene expression patterns. As part of RT-qPCR analysis, normalization of the data requires internal control gene(s) that display uniform expression under different biological conditions. However, no invariable internal control gene exists, and therefore more than one reference gene is needed to normalize RT-qPCR results. Identification of stable reference genes in potato will improve assay accuracy for selecting stress-tolerance genes and identifying molecular mechanisms conferring stress tolerance in this species.

**Results:**

In the experiment, we assessed the expression of eight candidate internal control genes, namely elongation factor-1alpha (*EF1α*), *actin*, *tubulin*, glyceraldehyde-3-phosphate dehydrogenase (*GAPDH*), adenine phosphoribosyl transferase (*APRT*), 60S ribosomal protein L8 (*L8*), Cullin 3A (*CUL3A*), and exocyst complex component sec3 (*sec3*), in a diverse set of potato samples representing drought stress and osmotic stress challenges, and using geNorm, NormFinder, BestKeeper and RefFinder softwares.

**Conclusions:**

The results indicated that *EF1α* and *sec3* were the most stably expressed genes in the potato under drought and osmotic stress conditions. This work will facilitate future work on gene expression studies in potato and also benefit other species of the *Solanaceae*, such as tomato.

## Background

Drought and osmotic stress can seriously limit plant growth and productivity. Plants have developed multiple strategies to cope with drought and osmotic stress. These normally involve a mixture of stress avoidance and tolerance adaptations, which produce a range of changes at the morphological, physiological, cellular, and molecular levels [1]. Modern potato (*Solanum tuberosum* L.) varieties are considered to be sensitive to drought, but they have different morphological and physiological responses to water deficit compared to other crops [2]. Drought in field conditions results in significant losses in the yield and/or quality of potato tubers. With the increased global warming, there is a need to identify genotypes of the potato showing high tolerance to drought stress [3, 4].

Real-time quantitative PCR (RT-qPCR) is the most commonly used method for accurately detecting gene expression patterns [5]. It is necessary to use the normalization method for the variation of the control sample, caused by changes in RNA samples, reverse transcription efficiency and the quality of PCR efficiency. The use of one or more stable reference genes is the most common method for normalization of RT-qPCR data. As part of RT-qPCR analysis, normalization of the data requires internal control gene(s) that display uniform expression under different biological conditions [6]. However, no invariable internal control gene exists, and therefore more than one reference gene is needed to normalize RT-qPCR results [7]. Identification of stable reference genes in potato will improve assay accuracy for selecting stress-tolerance genes and identifying molecular mechanisms conferring stress tolerance in this species.

A reference gene is usually a housekeeping gene, ubiquitously expressed in all cells, and its product is essential for cell structure or metabolism, such as actin, ribosomal protein, *EF1*-*α*, and *GAPDH* [8]. However, these traditional reference genes are not always stably expressed under different circumstances. Therefore, it is necessary to select corresponding reference genes that are expressed at a constant level in certain cases. In potato, *EF1*-*α* was confirmed to be the most suitable reference gene under salt stress and late blight, and *EF1*-*α* and *APRT* were considered to be the most stable under cold stress [9, 10]. In addition, *sec3*, *CUL3A*, and *C2* were found to be most suitable for screening of reference genes in edible tubers [11]. However, the stability of these reference genes under other abiotic stresses has not been confirmed. To the best of our knowledge, there is no previous report on the selection of suitable reference genes for potato under drought and osmotic stress. Therefore, the selection of stable reference genes for potato is helpful for future molecular studies using RT-qPCR.

RNA-seq is a powerful technique that can be used to provide estimates of gene and/or transcript expression, and RPKM (Reads Per Kilobase per Million reads) is widely used to represent the relative abundance of mRNAs for a gene [12, 13]. In our previous study, we used RNA-sequenced potato samples to gain insight into the molecular basis of drought adaptation by comparing digital expression profiles between drought treatment and the control treatment [14]. To gain insight into the transcriptome dynamics that are associated with drought stress, genome-wide gene expression profile was conducted by Solexa sequencing to generate a large dataset and a comprehensive transcriptome profiling for potato. Finally, we identified a number of genes that were stably expressed under drought stress, including many recognized housekeeping genes. An effective method for selecting a stable expression of a reference gene candidate from the RNA-Seq data can be done by using the coefficient of variation (CV) [15]. However, the transcriptome with corresponding treatment is inherently limited; it cannot be used to determine a suitable reference gene(s) for other experimental conditions. Therefore, for a systematic selection of reference genes, RT-qPCR is still the primary approach [16].

In the present study, we selected eight stable genes of different metabolic pathways according to the previous RNAseq analysis and screened them as reference genes under drought and osmotic stress. The RT-qPCR data generated were analyzed using the three most widely used algorithms, namely geNorm, NormFinder and Bestkeeper, to determine sets of reference genes suitable for gene expression studies in different experimental conditions [17]. Additionally, a comprehensive reference gene stability analysis tool RefFinder was used to confirm the ranking results obtained from geNorm, NormFinder and Bestkeeper. This work will facilitate future work on gene expression studies in potato and also benefit other species of the *Solanaceae*, such as tomato.

## Methods

### Plant material, growth conditions and stress treatments

The experiments were carried out with the potato (*Solanum tubersosum* L.) tetraploid cultivar ‘Atlantic’. Potatoes were grown in pots of Gansu Agricultural University (Lanzhou, China) greenhouse facilities, maintained at 18–23 °C and 70% relative humidity under natural light conditions. Three replicate pots per treatment were arranged in a randomized block design. Drought stresses were applied to the 6-week-old plants by stopping irrigation in the treatment plots; the control plots were irrigated continuously. Fresh plants were collected from drought-treated potato plants every 5 days; plants were collected 6 times.

To prevent microbial contamination, potato shoots were removed from tubers, surface sterilized (ethanol, HgCl_2_ and sodium hypochlorite) and placed in 30% (w/v) sucrose Murashige-Skoog (MS) medium. The shoots were incubated at 20 °C for 20 days. The stem sections of the test-tube seedlings were cut and transplanted into a special culture tube with an opening at the bottom containing the new MS medium. When the plants were 6 cm long, replacement of MS medium containing 20% polyethylene glycol 6000 (C6 M), while the other three plants were grown under MS medium. Fresh leaves were collected after 0, 2, 4, 8, 16 and 32 h.

We also carry out simulated drought treatment as follows: some of the test tube seedlings were planted on quartz sand containing MS medium, and when the plants grew to 6 cm, the MS medium was removed from the bottom of the special culture tube.

Fresh leaves were collected at 0, 2, 4, 8, 12 and 32 days. All treated plant materials were immediately frozen in liquid nitrogen and stored at − 80 °C until further RNA extraction and target gene expression analysis.

### Potential reference gene selection

We selected 8 (*CUL3A*, *EF1α*, *GAPDH*, *sec3*, *tubulin*, *L8*, *APRT* and *actin*) commonly used reference genes for RT-qPCR analysis based on our previous publication [14] and our unpublished second RNAseq data set; these genes contain more than one exon. The sequences of these eight potato reference genes were obtained from the GenBank database and from the Potato Genomics Resource (http://solanaceae.plantbiology.msu.edu). Primer pairs were designed from these sequences with the NCBI Primer-BLAST program (Table 1) (http://www.ncbi.nlm.nih.gov/tools/primer-blast/) [18]. Primers were designed across exon boundaries to avoid genomic DNA contamination and exon analysis using Splign (http://www.ncbi.nlm.nih.gov/sutils/splign/splign.cgi) [11]. Before RT-qPCR analysis, PCR was performed using primers as shown in Table 1 to determine the size specificity of the amplicons, then electrophoresed on ethoxylated gels and ethidium bromide, and the target amplicons were sequenced to confirm the identity of the PCR product.

**Table 1:** Candidate reference genes and primer sequences

Gene	Gene code	Primer sequences (forward/reverse)	Amplicon length (bp)	log_2_(drought/CK)	Tm (°C)	E (%)	R^2^
*EF1α*	PGSC0003DMG400023270	GATGGTCAGACCCGTGAACA	106	0.148	60.9	102.95	0.999
		CCTTGGAGTACTTCGGGGTG					
*CUL3A*	PGSC0003DMG400001321	AGCATCGGGTTGTTGTGGAT	170	0.173	59.0	95.56	0.998
		TCCTGAATAGAGCTTCTCCCCA					
*GAPDH*	PGSC0003DMG400015253	GCTCATTTGAAGGGTGGTGC	151	0.257	58.8	101.69	0.997
		AGGGAGCAAGGCAATTTGTG					
*sec3*	PGSC0003DMG402015451	GCTTGCACACGCCATATCAAT	160	0.084	58.0	100.88	0.995
		TGGATTTTACCACCTTCCGCA					
*tubulin*	PGSC0003DMG400009938	GGGAATAACTGGGCGAAAGGT	134	− 0.185	60.0	97.00	0.996
		CCTCCACCAAGTGAGTGACAA					
*L8*	PGSC0003DMG400025015	GTTGGTAATGTGTTGCCGCT	172	0.328	58.8	102.96	0.996
		TGGCACCTGATGGGAGCTTA					
*APRT*	PGSC0003DMG400021527	CGTATCGCTGGGATTGCTTC	177	0.065	58.9	98.31	0.995
		TGCTTCAATACCTGCAACCAC					
*actin*	PGSC0003DMG400023429	AGGAGCATCCTGTCCTCCTAA	180	− 0.315	60.0	103.40	0.998
		CACCATCACCAGAGTCCAACA					

*log*_*2*_ *(drought/CK)* the log_2_ value of the ratio of drought treatment to control reads per kilo bases per million reads, *E* PCR efficiency, *Tm* annealing temperature, *R* ^*2*^ regression coefficient

### RNA extraction and first strand cDNA synthesis

The sampled plants were grounded to fine powder with mortar and pestle in liquid nitrogen, and 100 mg of the material was used for RNA isolation. Total RNA was extracted using Trizol TIANGEG (TIANGEN, Beijing, China), according to the manufacturer’s instructions. Isolated RNA was treated with RNase-free DNase (TaKaRa, Japan) to remove genomic DNA contamination. Purity and concentration of RNA samples were measured using a micro-volume UV spectrophotometer (Quawell Q5000, Quawell, USA) and integrity was checked on agarose gel electrophoresis. RNA samples with 260/280 ratio between 1.9 and 2.1 were used for subsequent experimentation. First strand cDNA synthesis using cDNA synthesis kit (Sangon, Shanghai, China) was performed according to manufacturer’s instructions in a total volume of 20 μl containing 2 μg total RNA. RNA extraction and cDNA synthesis from all samples were performed for three biological replicates. The cDNA solution was 10 times diluted with nuclease-free water and aliquots were stored at − 20 °C until use in RT-qPCR [19].

### Real-time quantitative PCR analysis

RT-qPCR analysis was carried out in 96-well plates with a Mx3005 multiplex quantitative RT-PCR system (Stratagene, USA) using the EvaGreen-based PCR assay. Each reaction (final volume of 20 µl) contained 1.0 μl template, 10.0 μl 2 ×  SuperReal Premix (TIANGEN, Beijing, China), 0.4  μl 50× ROX Reference Dye, 0.6  μl each primer (10 μmol/μl) and 6.2  μl double distilled water was carried out. The RT-qPCR conditions were pre-denaturation at 94  °C for 40 s, followed by 38 cycles of denaturation at 94  °C for 10 s, annealing at 57.2  °C for 30 s and with a final extension step at 72  °C for 10 min. Three technical replicates were set for each cDNA [20].

### Gene expression stability analysis

A standard curve, repeated in three independent plates using a tenfold serial dilution of the mixed cDNAs was obtained from all tested samples as templates. The correlation coefficients (R2) and slope values were acquired from the standard curve. Then, we calculated the gene-specific PCR amplification efficiency of each gene. The corresponding real-time PCR efficiencies were calculated according to the equation: ${\text{E}} = \left[ {10^{{\left( {{ - }1/slope} \right)}} - 1} \right] \times 100$ [21].

Simultaneously, the amplicon characteristics, including Tm, length, amplification efficiency with standard deviation, and correlation coefficient, of the eight candidate reference genes are listed in Table 1.

To compare stability of expression among the candidate reference genes, the computational methods, geNorm [22], Normfinder [23], and BestKeeper [24] were applied to quantification cycle (Cq) for each gene’s expression data. These tools are based on different models and assumptions and each produced different results for the same gene’s expression data [25]. RefFinder was used to calculate a recommended comprehensive ranking based on the results of computational analysis, which in turn allowed us to identify the best reference genes for RT-qPCR data normalization in potato samples [26].

For geNorm and NormFinder analysis, the raw Cq values under different experimental designs were transformed into relative quantities using the formula 2^−ΔCq^ (ΔCq = each corresponding Cq value-lowest Cq value) and then imported to geNorm to analyze gene expression stability value (M1). Similar to geNorm, NormFinder was further used to investigate the expression stability values (M2) for each gene and the pairwise variation of that gene with other reference genes. The reference gene with the highest M (M1 or M2) value is considered as the most unstable gene while the lowest M (M1 or M2) value indicated the most stable gene [22]. BestKeeper analysis was based on the untransformed Cq values and using coefficient of variance (CV) and the standard deviation (SD) of the Cq to evaluate the stability of reference genes. All three of the software programs were run based on the software manuals to select suitable reference genes [24]. By the combination of the three kinds of RefFinder (http://150.216.56.64/referencegene.php?type=reference) software, we could easily rank the expression stability of reference genes in different experimental sets [27].

## Results

### Assessment of primer specificity and PCR amplification efficiency

**Fig. 1:**
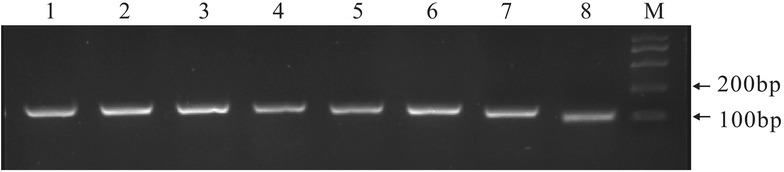
Specificity of PCR and amplicon length. Amplified fragments shown by agarose gel electrophoresis with ethidium bromide staining. *M* marker 2000. *Lanes 1*, *2*, *3*, *4*, *5*, *6*, *7* and *8* were the genes of *EF1α*, *tubulin*, *GAPDH*, *sec3*, *CUL3A*, *L8*, *APRT* and *actin* from potato

The sequences of *CUL3A*, *EF1α*, *GAPDH*, *sec3*, *tubulin*, *L8*, *APRT* and *actin* in potato were targeted by using specific primers on a template cDNA. The specificity of the designed primers was identified by gel electrophoresis and target amplicons were sequenced. The results showed a single band with the expected size by gel electrophoresis (Fig. 1), and the sequencing results of the target amplicon are also consistent with those of the target amplicons. The amplification efficiencies and correlation coefficients (R2) of 8 candidate reference genes in potato were calculated by slopes of the standard curves. The RT-qPCR amplification efficiencies for the 8 reference genes ranged from 95.56 to 103.40, and correlation coefficients ranged from 0.995 to 0.999 (Table 1). Thus, these primers can be used in RT-qPCR analysis.

### Cq Values of candidate reference genes

**Fig. 2:**
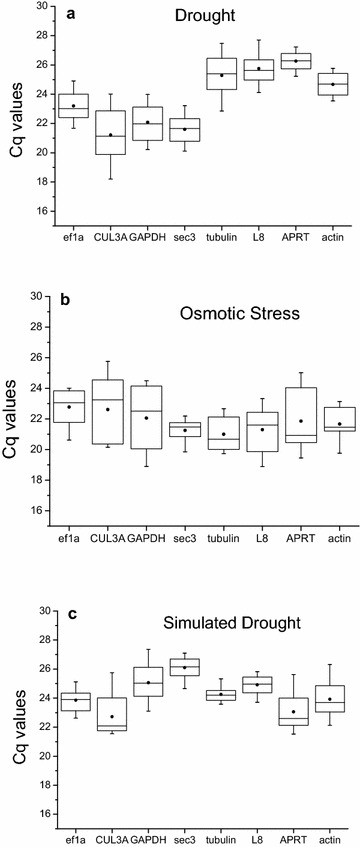
Expression levels of candidate reference genes in experimental samples. Expression data are displayed as Cq values for each reference gene in all samples. The line across the box indicates the median. The box indicates the 25 and 75th percentiles. Whiskers represent the maximum and minimum values. Points represent the average. **a** Drought, **b** osmotic, **c** simulated drought

The quantification cycle (Cq) values of the eight potential reference genes were assayed using RT-qPCR, with lower Cq values reflecting higher mRNA transcript levels. The Cq values of all the potential reference genes were between 17 and 30 under the three treatments (Fig. 2). The expression level of the potential reference gene was different under different treatments. *CUL3A* had the highest expression level under drought treatment (mean Cq of 21.2), while *APRT* had the lowest expression level (mean Cq of 26.3). Under the osmotic stress, *tubulin* had the highest expression level (mean Cq of 21.0), while *EF1a* had the lowest expression level (mean Cq of 22.8). *CUL3A* had the highest expression level under simulated drought (mean Cq of 22.7), while *sec3* had the lowest expression level (mean Cq of 26.1).

### Candidate reference genes expression stability

#### geNorm analysis

The gene expression stability of all the 8 candidate reference genes was evaluated using geNorm statistical algorithm. This software determines the normalization value based on the geometric mean of various candidate reference genes and mean pair wise variation of each gene from all the reference genes in a given set of samples. According to geNorm analysis the cut-off range of stability value (M1) is < 1.5, so the gene with lowest M1 value is considered to be the most stable reference gene in terms of gene expression, and vice versa. We analyzed our data for all three experimental sets and found that under drought and simulated drought all the 8 candidate reference genes exhibited high expression stability with low (< 0.8) M1 values, which were much lower than the default threshold of 1.5. However, only 5 genes are stable under osmotic stress, they are *EF1α*, *sec3*, *tubulin*, *L8*, and *actin*. Among all treatments, *EF1α* has the lowest M1 value followed by *sec3*, however *CUL3A* exhibited highest M1 value indicating that *EF1α* and *sec3* were most stable in expression and *CUL3A* the least (Table 2).

**Table 2:** Ranking of candidate reference genes based on stability values calculated by four softwares for three treatments

Approach	Gene	geNorm (M1)	Normfinder (M2)	BestKeeper (CV ± SD)	RefFinder
Drought stress	*EF1α*	0.375	0.079	3.93 ± 0.91	1.97
	*CUL3A*	0.772	0.520	6.93 ± 1.47	8.00
	*GAPDH*	0.417	0.105	4.79 ± 1.06	3.83
	*sec3*	0.395	0.130	3.49 ± 0.75	2.06
	*tubulin*	0.505	0.248	4.63 ± 1.17	6.19
	*L8*	0.432	0.151	3.30 ± 0.85	4.00
	*APRT*	0.580	0.376	1.96 ± 0.52	3.96
	*actin*	0.464	0.253	2.71 ± 0.67	2.78
Osmotic stress	*EF1α*	1.173	0.333	4.00 ± 0.91	2.06
	*CUL3A*	2.248	1.460	8.34 ± 1.89	8.00
	*GAPDH*	1.829	1.134	8.00 ± 1.77	6.48
	*sec3*	1.144	0.206	2.59 ± 0.55	1.57
	*tubulin*	1.482	0.773	4.43 ± 0.93	4.73
	*L8*	1.278	0.466	5.48 ± 1.17	2.99
	*APRT*	1.908	1.105	7.49 ± 1.64	6.48
	*actin*	1.226	0.129	3.69 ± 0.80	2.21
Simulated drought	*EF1α*	0.429	0.178	2.44 ± 0.58	1.86
	*CUL3A*	0.618	0.379	4.85 ± 1.10	8.00
	*GAPDH*	0.515	0.264	3.90 ± 0.98	5.73
	*sec3*	0.453	0.233	2.21 ± 0.58	2.06
	*tubulin*	0.562	0.337	1.62 ± 0.39	3.74
	*L8*	0.471	0.265	2.12 ± 0.53	2.63
	*APRT*	0.507	0.262	4.44 ± 1.02	5.60
	*actin*	0.468	0.214	4.03 ± 0.96	3.50

#### NormFinder analysis

The expression stability of 8 candidate genes was further analyzed using NormFinder software. NormFinder measures gene expression stability by comparing the variation within and between user-defined sample groups. Candidate control genes with lowest stability values have the minimum intra and intergroup variation and thus are top ranked. For each candidate gene, NormFinder provides a stability value (M2) that is a direct measurement of expression variation. Hence, it could easily be seen that *EF1α*, *actin* and *sec3* are the most stable reference genes for the three treatments. Among the most stable reference genes, *EF1α* had the lowest value which may be considered as the most important reference genes. More interestingly, *EF1α* has the lowest value under the three treatments consistent with the use of GeNorm analysis. However, there are also slight differences between the results of geNorm and NormFinder analysis. For instance, *actin* was considered as the most stable reference gene by geNorm under osmotic stress, while it was ranked third by NormFinder (Table 2).

#### BestKeeper analysis

BestKeeper calculates the BestKeeper Index from the geometric mean of the reference genes and performs Pearson correlation of each of the reference genes to the BestKeeper Index to indicate the correlation of that gene with the Index [28]. BestKeeper also calculates the standard deviation (SD) and the coefficient of variation (CV) based on the Cq values of all candidate reference genes [24]. Genes with SD greater than 1 are considered unacceptable. Reference genes are identified as the most stable genes, i.e. those that exhibit the lowest coefficient of variance and standard deviation (CV ± SD) [29]. The results of BestKeeper analysis are shown in Table 2. The best correlations were obtained for *APRT* (0.52), *actin* (0.67), *sec3* (0.75) and *L8* (0.85) in the drought stress, and for *sec3* (0.55), *actin* (0.80), *tubulin* (0.93) and *EF1α* (0.91) in the osmotic treatment, and for *tubulin* (0.39), *L8* (0.53), *sec3* (0.58) and *EF1α* (0.58) in the simulated drought treatment. Under the three treatments, the most unstable genes are identical to *CUL3A*.

#### RefFinder analysis

The results obtained from geNorm, NormFinder and BestKeeper were further confirmed using the comprehensive ranking platform RefFinder. RefFinder is a web-based tool which integrates three current computing programs to compare and re-rank the tested reference genes based on the geometric mean of the weights of every single gene calculated by each program. The final ranking results are shown in Table 2. For all the tested samples, a similar ranking order was obtained using RefFinder as compared to geNorm and NormFinder. Under drought treatment, the ranking order (from the most stable to the least stable) was: *EF1α* > *sec3* > *actin* > *GAPDH* > *APRT* > *L8* > *tubulin* > *CUL3A*. Under osmotic stress, the ranking order was: *sec3* > *EF1α* > *actin* > *L8* > *tubulin* > *GAPDH* > *APRT* > *CUL3A*. Under simulated drought treatment, the ranking order was: *EF1α* > *sec3* > *L8* > *actin* > *tubulin* > *APRT* > *GAPDH* > *CUL3A*.

Our combined results show, for normalization, *EF1α* and *sec3* exhibited the best stability under the three treatments, although their ranking was different. On the other hand *CUL3A* is the most unstable.

## Discussion

According to previous studies on the selection of plant reference genes for RT-qPCR, the expression level of a reference gene might not be constant across various species. Even the stable reference genes of tomato that are homologous to potato are also different [30, 31]. In addition, the expression of a reference gene can be different in the same species in response to various treatments or different plant tissues. For example, *EF1α* is considered to be the most stable reference gene under potato biotic (late blight) and abiotic stresses (salt stress) [9], but *EF1α* and *APRT* are the most stable genes under cold stress. In the potato tubers, the most suitable reference genes are *C2*, *sec3* and *CUL3A* [11].

In this study, 8 genes that have been commonly used as the candidate reference gene in many species were evaluated. Interestingly, *EF1a* and *sec3* exhibited good stability in potato under drought, osmotic and simulated drought treatments in this study. This indicates that there is an inherent link between the three processes. However, there are some differences in the rankings of potential reference gene stability. For example, the three most stable reference genes are *EF1α*, *sec3* and *actin* under drought stress, and the three most stable reference genes are *sec3*, *EF1α* and *actin*, while the three most stable reference gene of the simulated drought are *EF1α*, *sec3* and *L8*.

GeNorm, NormFinder, and BestKeeper are three programs based on statistical analysis. They are commonly used by researchers to assess the robustness of a gene that is used as a reference gene in RT-qPCR analysis. The operating principle of NormFinder is similar to that of the GeNorm program, but the latter can select suitable reference gene combinations and the optimal number of reference genes. In contrast to GeNorm and NormFinder, BestKeeper software directly makes calculations using Cq values [24]. In our analysis, the rankings created by GeNorm and NormFinder were similar, while the ranking obtained by the BestKeeper program was almost always different. A previous report revealed a similar difference between BestKeeper and other methods [32, 33]. The final rank of reference genes was determined with the RefFinder program, a web-based user-friendly comprehensive tool that integrates geNorm, Normfinder and BestKeeper [34]. Therefore, the use of multiple softwares in a comprehensive analysis will help to obtain a more accurate reference gene.

Finally, the ability to control water stress in plants is desirable for drought resistance studies. When plants are growing in soil, studies of the effects of water stress on physiology are usually either very short so that soil and plant water stress change little, or they involve repeated soil drying cycles. For many studies, however, water stress of soil-grown plants cannot be manipulated well enough for careful experiments. Therefore, the addition of osmotic agents, such as PEGs, sorbitol, or mannitol, to liquid nutrient media is very useful and has received considerable attention in the literature [35]. However, the toxicity of osmotic agents has also received increasing attention. PEG toxicity may come from toxic contaminants, and the other may be the accumulation of salt toxic levels. Therefore, we decided to look at additional drought simulation in this study; we planted the plant on quartz sand, and when the plant grows to 6 cm high, the culture medium is dried to avoid the use of osmotic regulators. It is worth noting that the stable reference gene under simulated drought conditions is similar to the other two treatments. In summary, in this study, we analyzed the stability of reference genes for RT-qPCR in potatoes under drought and osmotic stress conditions. The combination of *EF1α* with *sec3* was suitable for gene quantification in potato under drought and osmotic stress. This study also proved that the drought formed by the quartz sand culture has a high degree of similarity in the internal reference gene.

## Conclusion

This study represents the first attempt to select a set of commonly used candidate reference genes in potato under drought and osmotic stress for the normalization of gene expression data using RT-qPCR. We showed that the most suitable reference gene is *EF1α* in drought and simulated drought environments, and the most suitable gene under osmotic stress is *sec3*. While different pairs were found to be the most appropriate for these biological contexts, we observed that the *EF1α* and *sec3* showed the most stable under all three treatments.

## References

[1] Zhu JK (2016). Abiotic stress signaling and responses in plants. Cell.

[2] Sprenger H, Kurowsky C, Horn R, Erban A, Seddig S, Rudack K (2016). The drought response of potato reference cultivars with contrasting tolerance. Plant, Cell Environ.

[3] Penuelas J, Sardans J, Estiarte M, Ogaya R, Carnicer J, Coll M (2013). Evidence of current impact of climate change on life: a walk from genes to the biosphere. Glob Chang Biol.

[4] Vasquez-Robinet C, Mane SP, Ulanov AV, Watkinson JI, Stromberg VK, De Koeyer D (2008). Physiological and molecular adaptations to drought in Andean potato genotypes. J Exp Bot.

[5] Livak KJ, Schmittgen TD (2001). Analysis of relative gene expression data using real-time quantitative PCR and the 2(-Delta Delta C(T)) method. Methods.

[6] Bustin SA, Benes V, Garson JA, Hellemans J, Huggett J, Kubista M (2009). The MIQE guidelines: minimum information for publication of quantitative real-time PCR experiments. Clin Chem.

[7] Udvardi MK, Czechowski T, Scheible WR (2008). Eleven golden rules of quantitative RT-PCR. Plant Cell.

[8] Czechowski T, Stitt M, Altmann T, Udvardi MK, Scheible WR (2005). Genome-wide identification and testing of superior reference genes for transcript normalization in *Arabidopsis*. Plant Physiol.

[9] Nicot N, Hausman JF, Hoffmann L, Evers D (2005). Housekeeping gene selection for real-time RT-PCR normalization in potato during biotic and abiotic stress. J Exp Bot.

[10] Lopez-Pardo R (2013). Selection of housekeeping genes for qRT-PCR analysis in potato tubers under cold stress. Mol Breed.

[11] Mariot RF, de Oliveira LA, Voorhuijzen MM, Staats M, Hutten RC, Van Dijk JP (2015). Selection of reference genes for transcriptional analysis of edible tubers of potato (*Solanum tuberosum* L.). PLoS ONE.

[12] Mortazavi A, Williams BA, McCue K, Schaeffer L, Wold B (2008). Mapping and quantifying mammalian transcriptomes by RNA-Seq. Nat Methods.

[13] Fonseca NA, Marioni J, Brazma A (2014). RNA-Seq gene profiling–a systematic empirical comparison. PLoS ONE.

[14] Zhang N, Liu B, Ma C, Zhang G, Chang J, Si H (2014). Transcriptome characterization and sequencing-based identification of drought-responsive genes in potato. Mol Biol Rep.

[15] Yim AK, Wong JW, Ku YS, Qin H, Chan TF, Lam HM (2015). Using RNA-Seq data to evaluate reference genes suitable for gene expression studies in soybean. PLoS ONE.

[16] Gantasala NP, Papolu PK, Thakur PK, Kamaraju D, Sreevathsa R, Rao U (2013). Selection and validation of reference genes for quantitative gene expression studies by real-time PCR in eggplant (*Solanum melongena* L.). BMC Res Notes.

[17] Wang Q, Ishikawa T, Michiue T, Zhu BL, Guan DW, Maeda H (2012). Stability of endogenous reference genes in postmortem human brains for normalization of quantitative real-time PCR data: comprehensive evaluation using geNorm, NormFinder, and BestKeeper. Int J Legal Med.

[18] Sivakumar T, Lan DT, Long PT, Yoshinari T, Tattiyapong M, Guswanto A (2013). PCR detection and genetic diversity of bovine hemoprotozoan parasites in Vietnam. J Vet Med Sci.

[19] Li X, Zhang D, Li H, Gao B, Yang H, Zhang Y (2015). Characterization of reference genes for RT-qPCR in the desert moss *Syntrichia caninervis* in response to abiotic stress and desiccation/rehydration. Front Plant Sci.

[20] Kundu A, Patel A, Pal A (2013). Defining reference genes for qPCR normalization to study biotic and abiotic stress responses in *Vigna mungo*. Plant Cell Rep.

[21] Kong Q, Yuan J, Gao L, Zhao S, Jiang W, Huang Y (2014). Identification of suitable reference genes for gene expression normalization in qRT-PCR analysis in watermelon. PLoS ONE.

[22] Vandesompele J, De Preter K, Pattyn F, Poppe B, Van Roy N, De Paepe A (2002). Accurate normalization of real-time quantitative RT-PCR data by geometric averaging of multiple internal control genes. Genome Biol.

[23] Andersen CL, Jensen JL, Orntoft TF (2004). Normalization of real-time quantitative reverse transcription-PCR data: a model-based variance estimation approach to identify genes suited for normalization, applied to bladder and colon cancer data sets. Cancer Res.

[24] Pfaffl MW, Tichopad A, Prgomet C, Neuvians TP (2004). Determination of stable housekeeping genes, differentially regulated target genes and sample integrity: BestKeeper–Excel-based tool using pair-wise correlations. Biotechnol Lett.

[25] Long X, He B, Gao X, Qin Y, Yang J, Fang Y (2015). Validation of reference genes for quantitative real-time PCR during latex regeneration in rubber tree. Gene.

[26] Huang L, Yan H, Jiang X, Yin G, Zhang X, Qi X (2014). Identification of candidate reference genes in perennial ryegrass for quantitative RT-PCR under various abiotic stress conditions. PLoS ONE.

[27] Chen Y, Hu B, Tan Z, Liu J, Yang Z, Li Z (2015). Selection of reference genes for quantitative real-time PCR normalization in creeping bentgrass involved in four abiotic stresses. Plant Cell Rep.

[28] De Spiegelaere W, Dern-Wieloch J, Weigel R, Schumacher V, Schorle H, Nettersheim D (2015). Reference gene validation for RT-qPCR, a note on different available software packages. PLoS ONE.

[29] Guo J, Ling H, Wu Q, Xu L, Que Y (2014). The choice of reference genes for assessing gene expression in sugarcane under salinity and drought stresses. Sci Rep.

[30] Lovdal T, Lillo C (2009). Reference gene selection for quantitative real-time PCR normalization in tomato subjected to nitrogen, cold, and light stress. Anal Biochem.

[31] Muller OA, Grau J, Thieme S, Prochaska H, Adlung N, Sorgatz A (2015). Genome-wide identification and validation of reference genes in infected tomato leaves for quantitative RT-PCR analyses. PLoS ONE.

[32] Mallona I, Lischewski S, Weiss J, Hause B, Egea-Cortines M (2010). Validation of reference genes for quantitative real-time PCR during leaf and flower development in *Petunia hybrida*. BMC Plant Biol.

[33] Rapacz M, Pień AS, Skorupa K (2012). Internal standards for quantitative RT-PCR studies of gene expression under drought treatment in barley (*Hordeum vulgare* L.): the effects of developmental stage and leaf age. Acta Physiol Plant.

[34] Janska A, Hodek J, Svoboda P, Zamecnik J, Prasil IT, Vlasakova E (2013). The choice of reference gene set for assessing gene expression in barley (*Hordeum vulgare* L.) under low temperature and drought stress. Mol Genet Genomics.

[35] Lagerwerff JV, Ogata G, Eagle HE (1961). Control of osmotic pressure of culture solutions with polyethylene glycol. Science.

